# Case Report: Substance fixation in autism spectrum disorder with resultant anorexia nervosa

**DOI:** 10.3389/fpsyt.2025.1630528

**Published:** 2025-09-23

**Authors:** Lucas Arney, Raymond Uymatiao, Justin White

**Affiliations:** ^1^ Virginia Tech Carilion School of Medicine, Roanoke, VA, United States; ^2^ Psychiatry, Carilion Clinic, Roanoke, VA, United States

**Keywords:** case report, autism spectrum disorder, substance use disorder, anorexia nervosa, cannabis use disorder (CUD)

## Abstract

Autism spectrum disorder (ASD) has a long-standing history of being strongly associated with multiple psychiatric comorbidities, including substance use disorders (SUDs) and eating disorders (EDs) like anorexia nervosa (AN). ASD-specific features, including repetitive behaviors, constricted interests, cognitive rigidity, and obsessive fixations, are believed to make patients with ASD vulnerable to SUD and ED development. However, little research attempts to understand the interconnection between ASD and these comorbidities. We present a case of a 26-year-old man with a long-standing diagnosis of ASD who developed SUD and restrictive food intake, culminating in the patient meeting the AN criteria. This patient voluntarily admitted himself to the inpatient psychiatric unit with worsening depression and suicidal ideation following a car accident resulting from sleep deprivation and acute cannabis intoxication. Further investigation revealed a problematic, obsessive pattern of initial alcohol use associated with weight gain, followed by profound food restriction and subsequent transition to daily, near-constant cannabis use. On presentation, he appeared anxious with overt signs of sleep deprivation and malnutrition from substantial weight loss with a body mass index (BMI) decline from 23.6 to 16.98 over the last year. A urine drug screen was positive for cannabinoids, and imaging was unremarkable. Over an 11-day hospitalization, a multidisciplinary team initiated anxiolytics, antidepressants, sleep aids, and cannabis-withdrawal management combined with nutritional rehabilitation under dietitian supervision until acute suicidality was resolved with an improved BMI to 18.75. At discharge, the patient reported eagerness to engage with outpatient psychotherapy, ongoing psychiatric follow-up, and ASD-adapted cognitive behavioral therapy. This case underscores the diagnostic and management implications associated with co-occurring ASD, SUD, and ED. Obsessive fixation and rigidity potentiate maladaptive coping, which, if unaddressed in therapy, may increase the risk of future SUD and ED relapse. Though rapid inpatient stabilization is possible, gaps in both specialty care and ASD-adapted programs may compound relapse risk, especially in underserved regions. Additionally, this case necessitates a comprehensive assessment of patients with neurodevelopmental disorders for more informed and integrated therapeutic intervention. The nuanced interplay between ASD, SUD, and ED has synergistic effects on caloric restriction, requiring multidisciplinary treatment strategies to achieve sustained recovery and reduce morbidity in a vulnerable population.

## Introduction

Autism spectrum disorder (ASD) is defined by the DSM-5-TR as a neurodevelopmental condition with deficits in social communication and interaction, alongside the presence of “restricted, repetitive patterns of behavior, interests, or activities” (1). Recent literature has increasingly shown that patients with ASD are at an elevated risk for developing numerous psychiatric comorbidities—attention deficit/hyperactivity disorder (ADHD), depression, anxiety, substance use disorder (SUD), and eating disorders (2–5). Specifically, a large 2025 meta-analysis highlighted that 29% of individuals with anorexia nervosa (AN) met the diagnostic criterion for ASD (6). Additionally, ASD patients generally are at twice the risk of developing SUD while also having three times the risk of bullying victimization compared to the general population (7–9). With established SUD risk factors of depression, anxiety, ADHD, and a history of experiencing violence, patients with comorbid ASD may be at an even greater than two-fold risk for developing SUD (10, 11).

**Figure 1:**
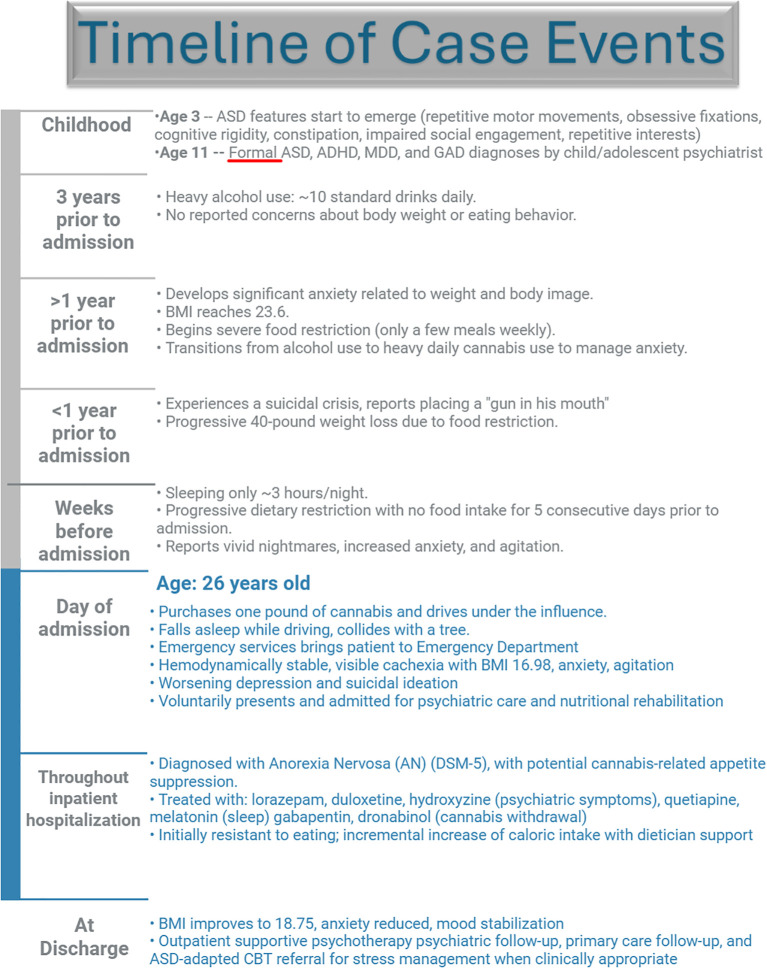
Patient timeline.

SUD is defined by the DSM-5-TR as a problematic pattern of use of an intoxicating substance with cravings, unsuccessful efforts to control substance use, and continued use despite recurrent negative implications (1). Substances can help fulfill the desire of a patient with ASD to fit in while decreasing anxiety. Specifically, our patient with established ASD struggled with alcohol use disorder (AUD) and cannabis use disorder (CUD). Both of these substances temporarily decrease anxiety but can induce heightened anxiety during withdrawal (12, 13). This case report will discuss this patient’s multiple psychiatric conditions and their relation to his ASD (**Figure 1**).

## Case description

A 26-year-old man with a past medical history of ASD, ADHD, GAD, MDD, AUD, and CUD was voluntarily admitted to the inpatient psychiatric unit with worsening depression and suicidal ideation following a motor vehicle collision, secondary to falling asleep behind the wheel. After meeting his dealer to buy 1 lb of cannabis, the patient was driving home under the influence before veering off the road and colliding with a tree. Emergency medical services transported the patient to the emergency department, where a urine drug screen was positive for cannabinoids. Apart from chest abrasions with minor hypokalemia, the patient remained hemodynamically stable, while his physical exam, labs, and imaging were all grossly unremarkable. In describing the car accident, the patient gets visibly distraught with progressively increased anxiety and agitation. He reports notable sleep deprivation over the last few weeks, averaging only 3 h per night, with absolutely no food consumption over the last 5 days. Furthermore, he describes vivid nightmares, continually waking him up throughout the previous night.

A thorough diagnostic inpatient interview, supported by parental corroboration, revealed his recent dependence on various substances and their relation to his body weight (**Table 1**). The patient described daily consumption of approximately 10 standard drinks for 3 years before developing intense anxiety tied to his self-image. After reaching a BMI of 23.6, he became preoccupied with weight loss, leading to extreme food restriction, with consumption of only a couple of meals per week. He also transitioned from alcohol to chronic cannabis use to further limit caloric intake. Concerning his cannabis use, he relied on smoking as an anxiolytic “from the time he wakes up to the time he goes to sleep.” Additionally, through intricately detailed reports, he described the nuances of various cannabis strains, concluding by stating that his “DNA is made up of weed”. Over the past year, he lost approximately 40 lb due to persistent food restriction, reaching a BMI of 16.98 at admission.

**Table 1:** Initial mental status examination.

Appearance and constitutional	Well developed Malnourished Cachectic Grooming is normal with disheveled hair Dressed in a hospital gown, wearing a hat low on the forehead
Activity	No psychomotor agitation or slowing but animated gesturing with frequent hand movements, often while speaking Episodes of pacing across the room with gaze fixation and head down No involuntary movements or tics
Attitude	Cooperative and engaged in the interview Mildly guarded, particularly while exploring sensitive topics
Articulation/speech	Rate: increased Volume: increased Prosody: generally maintained with intermittent tone flattening
Orientation	Awake and alert to self, place, time
Concentration/attention	Easily distractable but capable of being redirected with prompting Concentration is sustained
Memory	Short-term grossly intact through presenting event recollection Long-term grossly intact through history recollection
Affect	Affect is anxious and intermittently tearful Range is from quiet, polite, and reserved to agitated and restless
Mood/congruency	“Anxious and unregulated” Affect is congruent with mood
Suicidality/homicidality	Suicidal ideation without a plan ▫ “I think about dying every day—I just want to drive off a cliff.” ▫ “There are many ways I’d kill myself but don’t have a specific plan.” No homicidal ideation
Thought content and perceptions	Visual/auditory hallucination: denied Does not appear to be reacting to internal stimuli Obsessive ruminations or compulsions: diet and nutrition-related preoccupations Delusions: body image dysmorphia
Thought processes	Logical, linear, and concrete Pronounced difficulty with abstract concepts and abstract reasoning, ▫ i.e., during the box-breathing technique, patient had difficulty understanding the interconnection between each line and a sustained breath
Fund of knowledge	Average for age and education
Language/vocab	Average for age and education
Insight	Has intellectual insight, verbally recognizing that his behavior has led to harm
Judgement	Poor, especially apparent through verbal discussion regarding stress management and evidenced by impulsivity of actions and commentary

Collateral information from his parents revealed a notable preoccupation with “food alternatives,” as evidenced by the patient’s YouTube feed. The mother also reported a critical, emotionally charged incident a year prior, where the patient put a “gun in his mouth” during an argument with his father. On review of systems, the patient endorsed anxiety, depression, suicidal ideation, insomnia, substance misuse (cannabis), malaise, and weight loss while being negative for audiovisual hallucinations, delusions, memory loss, and tics.

Per chart review, the patient has regular outpatient psychiatric follow-up with only two prescribed medications: methylphenidate for ADHD and fluoxetine for MDD. His diagnosis of ASD without intellectual or language impairment was officially established by a child psychiatrist at age 11, with symptoms of repetitive motor movements, obsessive fixations, constipation, impaired social engagement, insistence on routines, and intense fixed interests, which were noted as early as 3 years old. Both the patient’s psychiatrist and family practitioner describe concerns about substance use: alcohol use over the last 3 years and cannabis use over the last year. The patient had no established history of an ED nor previous psychiatric hospitalizations. Family psychiatric history is only notable for the maternal aunt having AUD and AN.

A comprehensive capacity assessment confirmed that the patient was able to understand, appreciate, and participate in clinical decision-making regarding his ongoing medical care.

Over the course of the patient’s hospitalization, the patient received scheduled lorazepam, duloxetine, and as needed, hydroxyzine to dissipate his severe anxiety and depression. Quetiapine and melatonin were given to help with sleep initiation and maintenance, and gabapentin with dronabinol was given to assist with cannabis withdrawal. As a result of meeting the DSM-5-TR diagnostic criteria, the patient was diagnosed with anorexia nervosa with a possible decrease in appetite secondary to chronic cannabis use and mood disturbance. While initially struggling to increase his food intake, over the 11-day hospital course and with the help of a dietitian, the patient increased his BMI to 18.75.

At discharge, the patient was without any thoughts of self-harm, overt mood or psychotic symptoms, or hallucinations. He displayed good insight into his presentation and was willing to engage with continuity of care. Outpatient services were arranged, including short-term counseling services for supportive psychotherapy, regular psychiatric follow-up to manage the patient’s medication regimen, and regular primary care monitoring for eating disorder relapse. Additionally, the patient and his family were provided with a local center referral for ASD-adapted cognitive behavioral therapy for stress management once stable enough to participate in meaningful behavioral work.

## Discussion

Unintentional injuries continue to be the leading cause of death in the USA under 45 years old (14, 15). At 80,391 deaths in 2024, drug overdose remains the single largest contributor despite a historic 26.9% decline since 2023. This trend highlights the persistent need for effective SUD surveillance and risk factor identification, especially in vulnerable populations like those with ASD.

Prior research has linked SUD to impulsivity, and while ASD is highly comorbid with ADHD, ASD has additional features that contribute to substance use vulnerability (16). This case report underscores the role of these ASD-specific features, including repetitive behaviors, constricted interests, cognitive rigidity, and obsessive fixations (4, 7). Our patient describes cannabis use as a means to ameliorate overwhelming anxiety, stating that smoking became habitual over the course of his day and became “a part of his DNA,” emphasized as an obsession by the patient’s parents. This patient’s report proves consistent with other narratives and investigations described across the literature, which link ASD sensory dysregulation and anxiety to hazardous substance use (17, 18).

SUD can have numerous effects on the body, depending on the specific substance utilized. In this case, our patient with established ASD initially struggled with hazardous alcohol use and experienced weight gain, which was followed by strict food restriction, a transition to chronic cannabis use, and ultimately met the criteria for AN. Current literature highlights that chronic cannabis use often induces appetite suppression, especially in ASD populations (19, 20). Combined with a primary eating disorder and worsening depression, it is believed that CUD contributed to the patient’s ability to withstand his food drive. Alternative explanations include 1) a predominant depression-related appetite change, given the recent history of worsening mood symptomology; 2) an anxiety-driven food restriction, where food is established as a locus of control among a tumultuous familial environment; and 3) an underreported baseline ASD-related food aversion secondary to strict meal regimens and sensory dysfunction.

AN is defined by the DSM-5-TR as having a distorted body image that results in excessive dieting and severe weight loss with a pathological fear of gaining weight (1). With the genetic susceptibility initially described four decades ago (21), recent literature documents neuropsychological and behavioral overlaps between AN and ASD, such as dysregulated emotional recognition, social–emotional regulation, and emotional introspection (22, 23). Additionally, ASD is being increasingly recognized as a frequent comorbidity in eating disorder treatment facilities, a notable concern given that ASD-AN patients report unique challenges, often not amenable to standard eating disorder treatment regimens (24–26). From individualized therapy to diet plan adaptation for sensory-related accommodations, specific techniques are required in optimizing the overlap in outpatient treatment (27). Patients, like ours, are not likely to break a negative association with food during their inpatient stay. This goal is worked toward through months of ASD-adapted outpatient therapy, as the mainstay for reducing relapse risk.

This case report is limited by various factors. First, this is a single case report of a patient’s 11-day inpatient psychiatric hospitalization, reducing generalizability to broader patient populations. Second, minimal longitudinal follow-up restricts our ability to assess treatment efficacy beyond hospitalization. Third, broad systemic remarks on regional specialty care shortages are not directly supported by this case and are merely presented as potential considerations for future directions, rather than evidence-backed recommendations.

In conclusion, this case highlights the critical importance of recognizing specific ASD features as potential additive risk factors for SUD and ED development. Primary care physicians and psychiatrists must routinely monitor their ASD patients for SUD and ED, especially during periods of emotional distress and functional decline. Obsessive fixations and cognitive rigidity may potentiate maladaptive coping behaviors that lead to SUD and ED development or relapse. If hospitalized following relapse, a multidisciplinary, neurodiversity-informed approach from psychiatry, dietetics, ASD specialists, and patient families is required for the development of individualized treatment plans sensitive to each patient’s behavioral patterns and sensory profile. Additionally, proactive discharge planning with early integration of ASD-adapted CBT is necessary to augment coping skills and address cognitive rigidity. For the field as a whole, significant gains will come from large-scale epidemiological and prospective longitudinal cohort studies of ASD populations, which are needed to define the true prevalence, incidence, and outcomes of overlapping ASD–SUD–ED. Subgroup analyses, based on autism severity, sensory and emotional dysregulation, rigidity, and obsessive traits, would provide a more comprehensive understanding of the interplay between ASD features and relapse susceptibility. Lastly, the development and validation of neurodiversity-affirming care models may help bridge systemic gaps and promote equitable treatment in this vulnerable patient group.

## Data Availability

The original contributions presented in the study are included in the article/supplementary material. Further inquiries can be directed to the corresponding author.
